# Comparative in Vitro Cytotoxicity of Realistic Doses of Benchmark Multi-Walled Carbon Nanotubes towards Macrophages and Airway Epithelial Cells

**DOI:** 10.3390/nano9070982

**Published:** 2019-07-06

**Authors:** Luisana Di Cristo, Massimiliano G. Bianchi, Martina Chiu, Giuseppe Taurino, Francesca Donato, Giacomo Garzaro, Ovidio Bussolati, Enrico Bergamaschi

**Affiliations:** 1 Laboratory of General Pathology, Department of Medicine and Surgery, University of Parma, 43125 Parma, Italy; 2 Department of Public Health Sciences and Pediatrics, University of Turin, 10126 Turin, Italy

**Keywords:** airway epithelium, barrier permeability, inflammation, macrophages, multiwalled carbon nanotubes, realistic doses

## Abstract

Multi-walled carbon nanotubes (MWCNT) have many outstanding physical and chemical properties that make them useful in many applications in nanotechnology. However, these properties are reported to be potentially harmful for the human body. The effects of low and realistic doses of three well-characterized preparations of MWCNT, obtained from the Joint Research Centre (JRC) (NM-400, NM-401, and NM-402), were assessed in two murine macrophage lines, Raw264.7, of peritoneal origin, and MH-S, derived from alveolar macrophages. Macrophage viability, evaluated with two distinct methods, was significantly lowered by NM-401 (needle-like, average length 4 μm, diameter 67 nm) with IC_50_ values of 10 μg/cm^2^, whereas NM-400 and NM-402 (tangled, average lengths 846–1372 nm, diameter 11 nm) had much smaller effects. In contrast, at 10 μg/cm^2^, NM-400 and NM-402 induced the M1 marker *Nos2* and, consistently, a sizable accumulation of nitrites in the medium, whereas NM-401 had no significant effect. None of the MWCNT preparations induced the M2 marker *Arg1*. Phagocytic activity, assessed in Raw264.7 macrophages, was significantly reduced in cells exposed to NM-401, but not to NM-400 or NM-402. When tested on Calu-3 bronchial epithelial cell monolayers, the three MWCNT preparations did not affect cell viability, but decreased the trans-epithelial electrical resistance at the maximal dose tested (80 μg/cm^2^), with the most evident effect detected for NM-401, even at 10 μg/cm^2^. In conclusion, among the possible structural determinants of the toxic effects exerted by MWCNT towards macrophages and airway epithelial cells, shape and length appear the most relevant at low, realistic doses.

## 1. Introduction

Since their discovery [1,2], multi-walled carbon nanotubes (MWCNT) have attracted much interest owing to their excellent physicochemical, electrical, and mechanical properties and are increasingly used in a wide array of applications, such as microelectronics, energy storage, and biomedicine [3]. The ever-growing interest in these nanomaterials has favored the synthesis of an uncountable variety of preparations, leading to a very high degree of heterogeneity in MWCNT of either industrial or laboratory origin. Thus, every MWCNT preparation has peculiar features, such as length, diameter, aspect ratio, shape, purity, surface area, and aggregation/agglomeration tendency, each of which is potentially involved in determining the interaction between the nanomaterials and the biological systems. Indeed, respirable MWCNT agglomerates may be generated during various manufacturing steps, as a result of transfer, weighing, blending, or disposal [4]. For this reason, inhalation is considered the primary route for human exposure to MWCNT, especially in occupational settings [5]. As a consequence, the toxicological evaluation of each MWCNT preparation would require a case-by-case study, an evidently unfeasible task. Thus, a strong effort for MWCNT categorization has been undertaken, so as to simplify the toxicological assessment and, possibly, the enforcement of regulatory rules. However, although there has been a steady progress in the field, summarized in several excellent reviews [6,7,8,9], lack of a clear-cut identification of toxicity determinants does not yet allow a definite grouping of MWCNT.

Most in vitro studies aimed at describing the biological consequences induced by MWCNT are carried out using relatively high doses, which are far larger than those potentially reached under in vivo exposure conditions [10]. Consequently, there is a clear need to overcome this limitation using relevant doses for the occupational settings, in accordance with the permissible exposure limit of 1 μg/m^3^, suggested by the National Institute for Occupational Safety and Health (NIOSH) [11], an approach increasingly applied in recent studies.

The Joint Research Centre (JRC) Nanomaterial Repository is the first collection of thoroughly-characterized nanomaterials of industrial origin available for benchmarking in research and regulatory studies (https://ec.europa.eu/jrc/en/scientific-tool/jrc-nanomaterials-repository). As far as MWCNT are concerned, the materials available in the JRC Repository have been already investigated in several projects and studies [12,13,14,15,16,17,18]. However, a direct comparison of these benchmark MWCNT under the same experimental conditions, so as to identify possible toxicity determinants, has been performed rarely. In particular, as far as in vitro assays are concerned, Louro et al. [17] have compared the cytotoxic and genotoxic effects of four JRC MWCNT preparations in two airway epithelial cell lines so as to correlate the biological effects detected with the physico-chemical features of the MWCNT samples. More recently, benchmark MWCNT have been used in vivo to study the acute phase response following pulmonary exposure in mice [19].

However, it is only partially appreciated that for MWCNT, as for other nanomaterials, the outcome of the interaction with biological systems depends not only on the characteristics of the nanomaterial, but also on those of the biological system itself. Thus, the relevance of a specific physico-chemical feature may be different in different cells or tissues. For example, the interaction between nanomaterials (NM) and macrophages is markedly influenced by the ability of the phagocyte to internalize the nanomaterial or its agglomerates, which, in turn, is highly dependent on the size of the NM particles or of their agglomerates. This interaction is of paramount importance, since it can contribute to determining if the exposure to the nanomaterial will be followed by an inflammatory response [20,21].

In this work, part of a European Project aimed at managing the risk derived from the exposure to nanomaterials, macrophages, acting as the first line of immunological defense, were selected as case-study cell models. We have evaluated the effects on macrophages of three benchmark MWCNT derived from the JRC Repository. The evaluated endpoints included cell viability, the production of nitric oxide (NO), and the expression of inflammation-related genes, as markers of pro-inflammatory activity. Moreover, taking into account the possibility of unwanted inhalation at the occupational level, we have extended the study to a model of bronchial epithelial cells, monitoring MWCNT-induced alterations in the trans-epithelial electrical resistance (TEER), a parameter not investigated by Louro et al. [17], which is a marker of the barrier competence of the epithelial monolayers.

## 2. Materials and Methods

### 2.1. Source and Characterization of MWCNT

The NM-400, NM-401, and NM-402 MWCNT preparations were provided by the JRC Nanomaterials Repository (Ispra, Varese, Italy). These nanomaterials are classified as representative test materials (RTM) and include random samples from one industrial production batch sub-sampled into vials under reproducible (Good Laboratory Practice, GLP) conditions, with the stability of the sub-samples monitored. They were used within the scope of the EU FP7 project “Managing risks of nanomaterials” (MaRiNa). The main physico-chemical characteristics of the MWCNT used in this study, all produced through carbon vapor deposition, are provided in a JRC Report [22] and summarized in Table 1.

MWCNT, pre-heated for 3 h at 230 °C to eliminate possible contaminations with lipopolysaccharide (LPS) and pre-wetted in 0.5% ethanol, were suspended (2.56 mg/mL) in Milli-Q water, supplemented with bovine serum albumin (BSA, 0.05%), and dispersed through sonication (16 min, 400 W with a Branson 5510 sonicator bath). Stock suspensions were briefly vortexed just after dilution with culture media for experiments.

No other synthetic surfactant agent was employed, as we decided to allow agglomeration so as to characterize MWCNT toxicological properties in conditions resembling a real-life environment [23]. The characterization of MWCNT agglomeration under conditions comparable to those adopted in this study has been performed by Tavares et al. [24] and by Louro et al. [17] who have demonstrated that, when suspended in culture medium, NM-401 form larger agglomerates than NM-400 and NM-402.

### 2.2. Cells and Experimental Treatments

Murine alveolar macrophages (MH-S), a gift of Prof. Dario Ghigo, University of Torino (Italy), were originally provided by the Cell Bank of the Istituto Zooprofilattico Sperimentale della Lombardia ed Emilia-Romagna (Brescia, Italy). Raw264.7 murine peritoneal macrophages and Calu-3 lung adenocarcinoma cells were also obtained from the same Cell Bank.

Both macrophage lines were routinely cultured in a humidified atmosphere of 5% CO_2_ in air in Falcon 10-cm diameter dishes (BD, Bioscience, San Josè, CA, USA) in RPMI1640 medium supplemented with 10% FBS, streptomycin (100 μg/mL)-penicillin (100 U/mL), L-glutamine (2 mM), and (for MH-S cells only) β-mercaptoethanol (0.05 mM). For treatments, cells were seeded in 96-well dishes (BD Biosciences) at a density of 1.5 × 10^4^ cells/well for viability experiments or in 24-well dishes (BD Biosciences) at a density of 1 × 10^5^ cells/well for nitrite determination and gene expression studies.

Calu-3 lung adenocarcinoma cells were cultured in EMEM supplemented with 10% FBS, 2 mM glutamine, 1 mM sodium pyruvate, and antibiotics. For the experiments, Calu-3 cells were seeded into cell culture inserts with membrane filters (pore size 0.4 μm) for Falcon 24-well-multitrays (Cat. No. 3095, Becton, Dickinson & Company, Franklin Lakes, NJ, USA), at a density of 75 × 10^3^ cells/cm^2^.

Doses of materials are expressed in μg/cm^2^ of monolayer; a fixed proportion was maintained between the culture surface and the volume of incubation, so as to maintain fixed the relationship between doses in μg/cm^2^ and doses in μg/mL (dose in μg/cm^2^ × 1.6 = doses in μg/mL).

### 2.3. Cell Viability

To evaluate the effects of the MWCNT preparations on cell viability, Raw264.7 and MH-S cells were treated with the NM preparations at the indicated doses, and cell viability was assessed after 24, 48, and 72 h of exposure using the resazurin [25] or the neutral red uptake (NRU) assays [26]. The resazurin assay was also performed on Calu-3 cell monolayers after 12 days of exposure, as described by Rotoli et al. [27]. Since nanomaterials could interfere with these assays, preliminary experiments were performed incubating either dye with MWCNT.

In particular, resazurin dye was incubated with all the MWCNT preparations tested at the maximal dose used in the viability experiments (80 μg/cm^2^) in the absence of cells, so as to evaluate autofluorescence; moreover, MWCNT were also added just before the reading, so as to assess possible fluorescence quenching. No interference was detected in either case (data not shown).

For NRU, the neutral red solution (in culture medium without phenol red) was incubated for 30 min with increasing doses of MWCNT or with the vehicle alone (0.05% BSA in water). At the end of the incubation, the mixture was centrifuged (300× *g* for 3 min), the supernatants resuspended in ethanol/acetic acid (neutral red destain solution), and the OD measured. No interference was found (data not shown).

### 2.4. Nitrite Concentration

Nitrite concentration in the medium was used as a proxy for NO production. Nitrites were measured with the fluorimetric method of Misko et al. [28], as modified by Di Cristo et al. [29]. Fluorescence (λ_ex_ 360 nm; λ_em_ 430 nm) was determined with the multimode plate reader Perkin Elmer Enspire.

### 2.5. Real Time PCR

Gene expression was monitored as described by Bianchi et al. [30] with modifications. Total RNA was isolated with the GenElute Mammalian Total RNA Miniprep Kit (Sigma-Aldrich, Milan, Italy). After reverse transcription, aliquots of cDNA from each sample were amplified in a total volume of 25 μL with the Go Taq PCR Master Mix (Promega Italia, Milan, Italy), along with the forward and reverse primers (5 pmol each) reported in Table 2.

Real-time PCR was performed in a 36-well RotorGeneTM3000, Version 5.0.60 (Corbett Research, Mortlake, Australia). For all the messengers to be quantified, each cycle consisted of a denaturation step at 95 °C for 20 s, followed by separate annealing (30 s) and extension (30 s) steps at a temperature characteristic for each pair of primers (see Table 2). Fluorescence was monitored at the end of each extension step. Melting curve analysis was added at the end of each amplification cycle. The analysis of the data was done according to the relative standard curve method [31]. Expression data are reported as the ratio between each investigated mRNA and *Gapdh* mRNA.

### 2.6. Phagocytic Activity

Fluorescent yellow-green polystyrene latex beads (2 μm, Sigma-Aldrich, Milano, Italy) were opsonized with 50% human serum for 30 min at 37 °C before the experiments. After the treatment in the presence of the MWCNT preparations, Raw264.7 macrophages were incubated for 2 h at 37 °C in complete growth medium in the presence of latex beads (20 microspheres/cell). Cell monolayers were then washed vigorously with PBS to remove extracellular beads, counterstained with 2 μM of the vital cytoplasmic dye CellTracker Red CMTPX (Invitrogen, Milano, Italy) and fixed with 2% paraformaldehyde for 10 min. The number of internalized latex particles was determined by counting intracellular fluorescent beads with a fluorescent microscope (Nikon, Tokyo, Japan). For each culture, at least 3–5 fields containing about 100–150 cells were analyzed. Phagocytic activity was calculated as the number of cells with at least one bead inside/total number of cells counted [32].

### 2.7. Trans-Epithelial Electric Resistance

Trans-epithelial electric resistance (TEER) was measured using an epithelial voltohmmeter (EVOM, World Precision Instruments Inc., Sarasota, FL, USA). For the experiments, Calu-3 cells were allowed to grow for 10–15 d until a tight monolayer was formed (TEER > 1000 Ω⋅cm^2^). NM were added in the apical chamber from stock solutions without changing the medium, and TEER was measured after 12 d. TEER changes were expressed as the percentage of the initial value adjusted for control cell monolayers according to Equation 1 [33]:

(1) $$TEER\;\left(\%\right)=\frac{Final\;TEER\;treated}{Final\;TEER\;control}\times \frac{Initial\;TEER\;control}{Initial\;TEER\;treated}$$

A blank value (resistance of the cell culture insert with medium without cells) was subtracted from each TEER value.

### 2.8. Statistics

Data are expressed as the means ± standard deviation (mean ± SD). Statistical analyses were performed with one-way ANOVA with the Bonferroni post hoc test or with a *t*-test for unpaired data. GraphPad Prism® software Version 6.00 (GraphPad Software Inc., San Diego, CA) was used. Results were considered significant at *p* < 0.05.

### 2.9. Reagents

Whenever not stated otherwise, all the reagents were provided by Sigma-Aldrich, Milan, Italy.

## 3. Results

### 3.1. Viability

**Figure 1:**
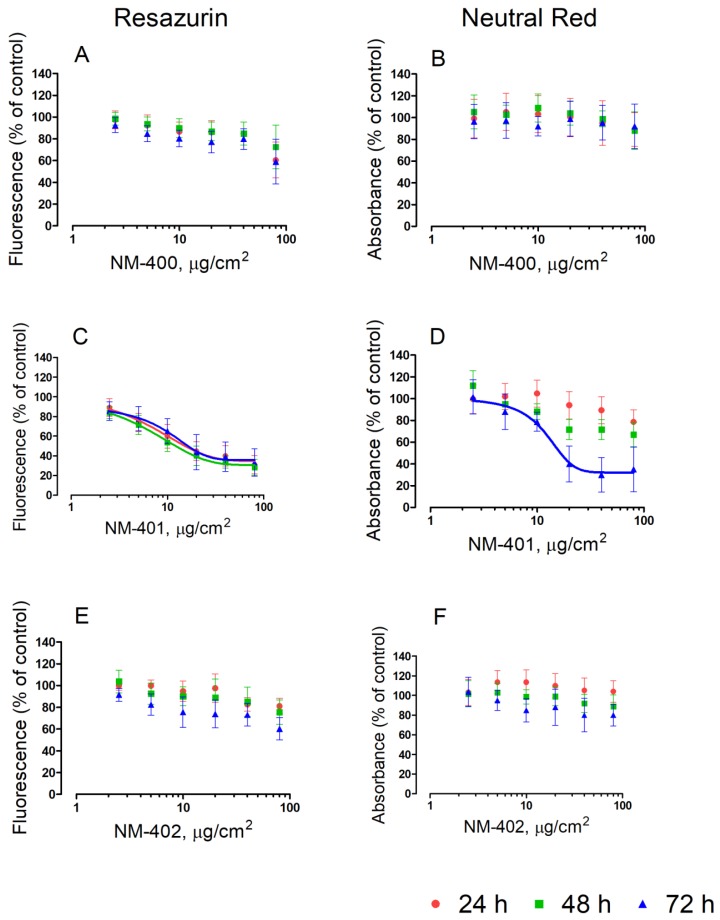
Effects of MWCNT on Raw264.7 murine macrophages. Cells were grown for 24 h in complete growth medium and then exposed for 24 h, 48 h, and 72 h to the indicated doses of MWCNT. At the end of the incubation, cell viability was assessed with resazurin (**A**,**B**,**C**) or neutral red uptake assays (**B**,**D**,**F**). Data are the means ± SD of five independent determinations. Lines represent the best fit to the dose-response curve (variable slope). For dose-response curves with NM-401, the R^2^values were 0.996, 0.999, and 0.993 at, respectively, 24 h, 48 h, and 72 h (resazurin); and 0.981 at 72 h (neutral red).

**Figure 2:**
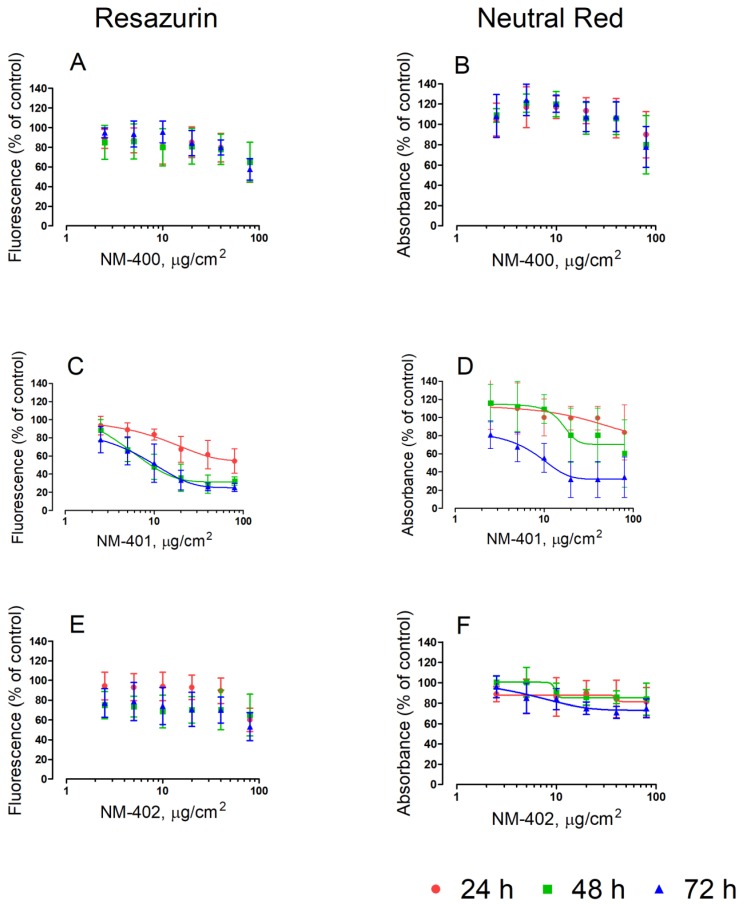
Effects of MWCNT on MH-S murine macrophages. Cells were grown for 24 h in complete growth medium and then exposed for 24 h, 48 h, and 72 h to the indicated doses of MWCNT. At the end of the incubation, cell viability was assessed with resazurin (**A**,**C**,**E**) or neutral red uptake assays (**B**,**D**,**F**). Data are the means ± SD of five independent determinations. Lines represent the best fit to the dose-response curve (variable slope). For the dose-response curves with NM-401, the R^2^ values were 0.990, 0.995, and 0.995 at, respectively, 24 h, 48 h, and 72 h (resazurin); and 0.936 and 0.972 at, respectively, 48 h and 72 h (neutral red).

The effects of the three MWCNT preparations on the viability of murine macrophages are shown in Figure 1 and Figure 2. Two different, independent methods, resazurin and neutral red uptake, were employed. With both methods, NM-400 and NM-402 caused a sizable decrease in the cell viability of Raw264.7 macrophages only at the highest dose tested (80 μg/cm^2^). On the contrary, cells underwent a marked, dose-dependent decrease in cell viability with the NM-401 preparation. At the longest time of incubation (72 h), macrophage viability decreased by 80%, as assessed with resazurin (IC_50_ = 10 μg/cm^2^), and by 70%, as assessed with neutral red uptake (IC_50_ = 11 μg/cm^2^), at the maximal dose adopted (Figure 1). Comparable results were obtained also with MH-S alveolar macrophages (Figure 2).

### 3.2. Macrophage Activation and Phagocytic Activity

**Figure 3:**
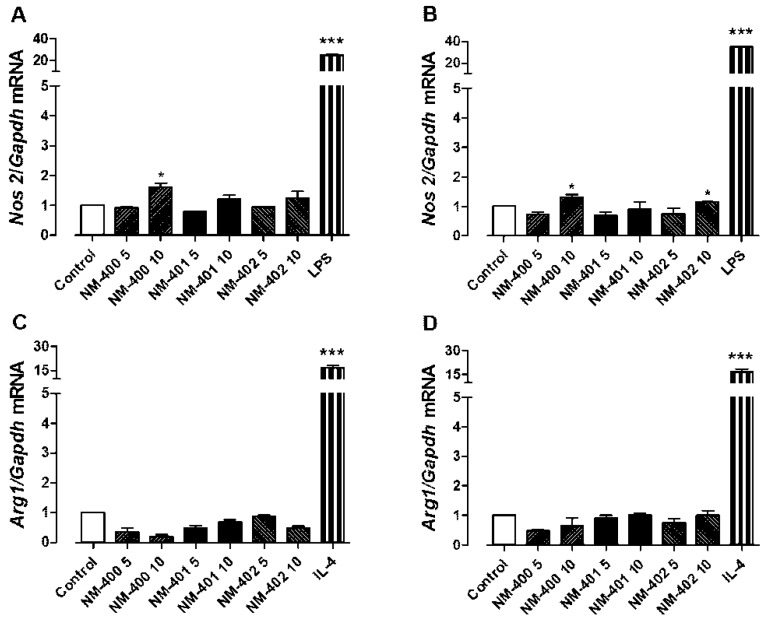
Nos2 and Arg1 expression in murine macrophages. Raw264.7 (**A**,**C**) and MH-S (**B**,**D**) macrophages, grown for 24 h in complete growth medium, were treated with 5 or 10 μg/cm^2^ of NM-400, NM-401, or NM-402 for 48 h. At the end of treatment, mRNA was extracted, and the expression of Nos2 (A,B) or Arg1 (C,D) was evaluated as described in the Materials and Methods. LPS (100 ng/mL) and IL-4 (10 ng/mL) were used as positive controls for, respectively, Nos2 and Arg1 induction. The experiment was performed twice with comparable results. Data are the means ± S.D. of two independent determinations, each performed twice. *p < 0.05 and **p < 0.01 vs. untreated, control cells, as assessed with the *t*-test for unpaired data.

**Figure 4:**
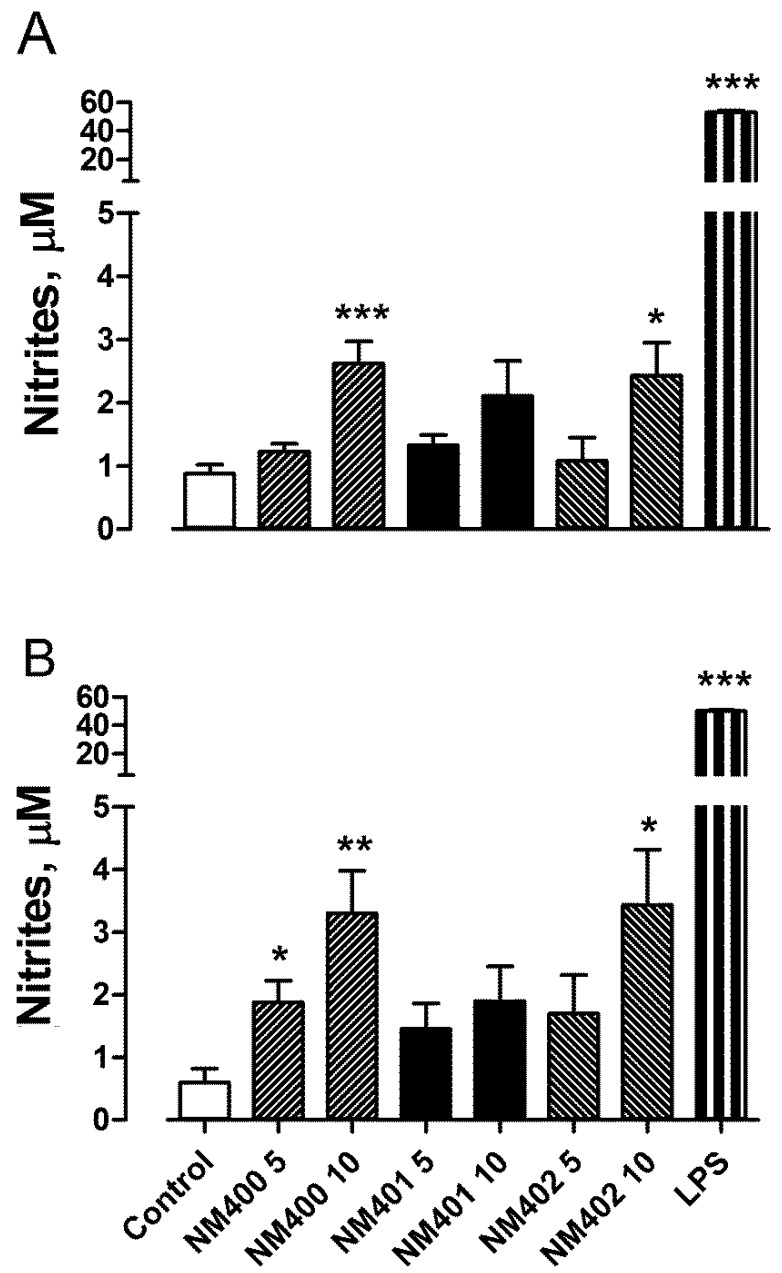
Effects of MWCNT on NO production in Raw264.7 and MH-S cells. Raw264.7 (**A**) and MH-S (**B**) macrophages, grown for 24 h in complete growth medium, were treated for 72 h with 5 or 10 μg/cm^2^ of NM-400, NM-401, or NM-402, or with LPS (100 ng/mL), used as a positive control. At the end of treatment, nitrite concentration was determined in the culture medium. Data are the means of seven independent determinations ± S.D. *p < 0.05, **p < 0.01, and ***p < 0.001 *vs.* untreated, control cells, as determined with the *t*-test for unpaired data.

The three preparations of MWCNT were also tested for their ability to trigger macrophage activation (Figure 3 and Figure 4). The expression of Nos2 and Arg1 (Figure 3) was assessed as markers of, respectively, M1 (classical) and M2 (alternative) murine macrophage activation. In Raw264.7 macrophages, only NM-400 at a dose of 10 μg/cm^2^ induced Nos2, while NM-401 and NM-402 were ineffective (Figure 3A,B). Furthermore, in MH-S cells, NM-400 induced Nos2 at 10 μg/cm^2^, but, in this cell model, a modest induction was also observed with NM-402 at the same dose, while exposure to NM-401 was without effect. None of the MWCNT preparations induced the M2 marker Arg1 in either macrophage line (Figure 3C,D).

Consistently with the data on gene expression, the determination of medium nitrite concentration (Figure 4), used as a proxy of NO production by murine macrophages [34], indicated that, after 72 h of exposure to MWCNT, NM-401 did not cause an increase in NO production by both macrophage types. On the contrary, at a dose of 10 μg/cm^2^, both NM-400 and NM-402 were able to induce a three-fold increase in NO output either in Raw264.7 or in MH-S cells.

**Figure 5:**
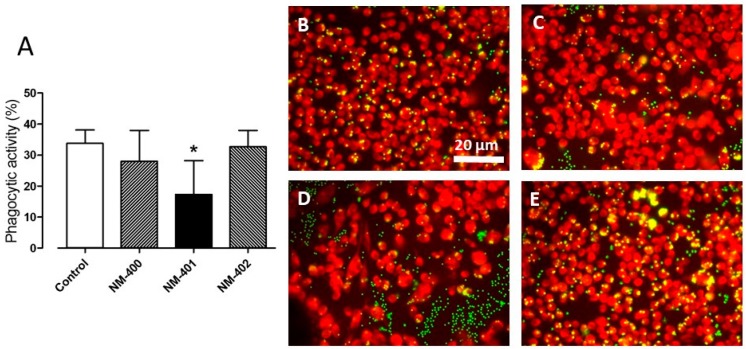
Effects of NM-400, NM-401, and NM-402 on the phagocytic activity. Raw264.7 macrophages were seeded on coverslips and, after a 24 h-exposure to 10 µg/cm^2^ of the indicated MWCNT, phagocytic activity determined as described in the Materials and Methods. (**A**) Data are expressed as the % of phagocytic activity and are the means ± SD (*n* = 3). * *p* < 0.05 vs. control cells, as determined with the *t*-test for unpaired data. (**B**–**E**) Images of representative fields taken with a fluorescence microscope. (**B**) Control, untreated cells. (**C)** NM-400-treated cells. (**D**) NM-401-treated cells. (**E**) NM-402-treated cells.

Both NM-400 and NM-402 did not cause significant impairment of the phagocytic activity of Raw264.7 macrophages at 10 μg/cm^2^; on the contrary, phagocytosis was roughly halved by NM-401 at the same dose (Figure 5).

### 3.3. Changes in Trans-Epithelial Electrical Resistance in Airway Cells

**Figure 6:**
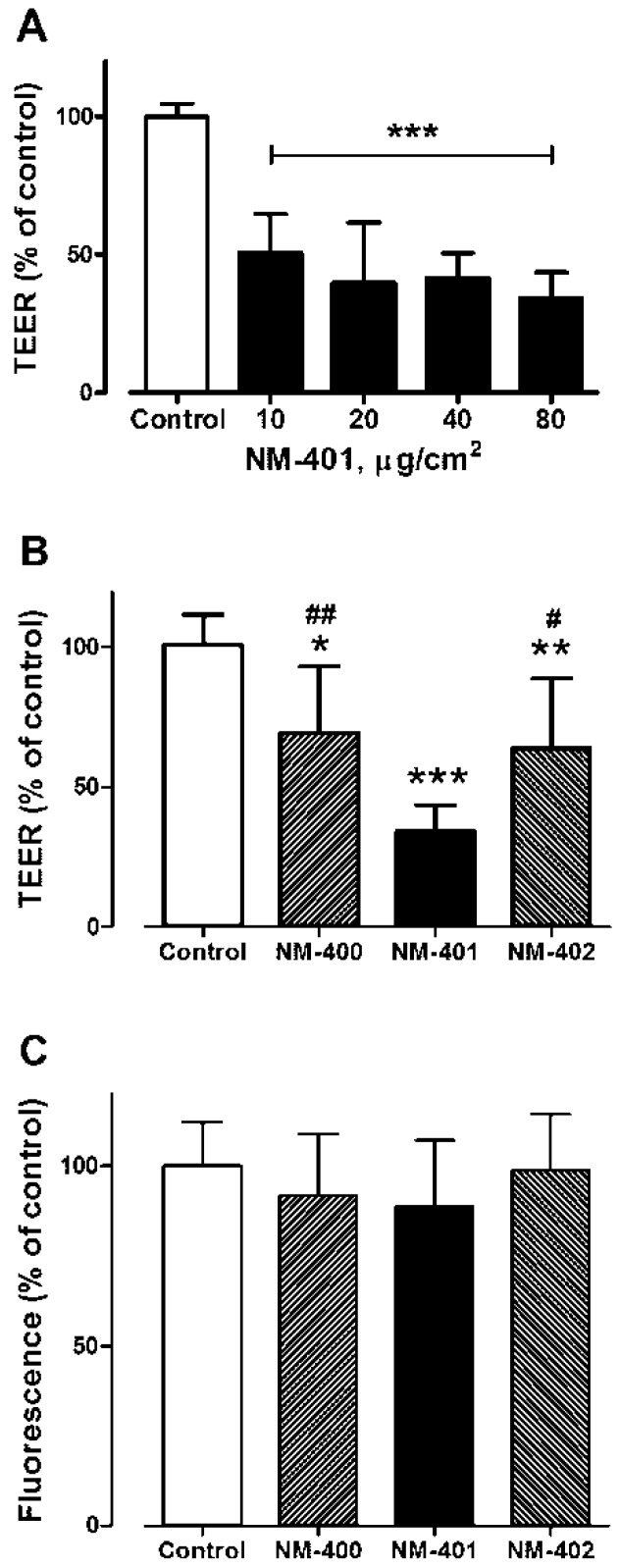
Effects of NM-400, NM-401, and NM-402 on the trans-epithelial electrical resistance (TEER) and viability of Calu-3 monolayers. Calu-3 cells were cultured for 10 days on 0.4-mm membrane filters. At the end of this period, MWCNT were added to the apical chamber of the culture system, at the doses indicated (**A**) or at 80 μg/cm^2^ (**B**,**C**). TEER (A,B) and viability (C) were determined after 12 days. Data are the means ± SD (*n* = 4). *, **, and *** *p* < 0.05, 0.01, and 0.001 vs. control, untreated cultures; # and ## *p* < 0.05 and 0.01 vs. monolayers treated with NM-401.

**Figure 7:**
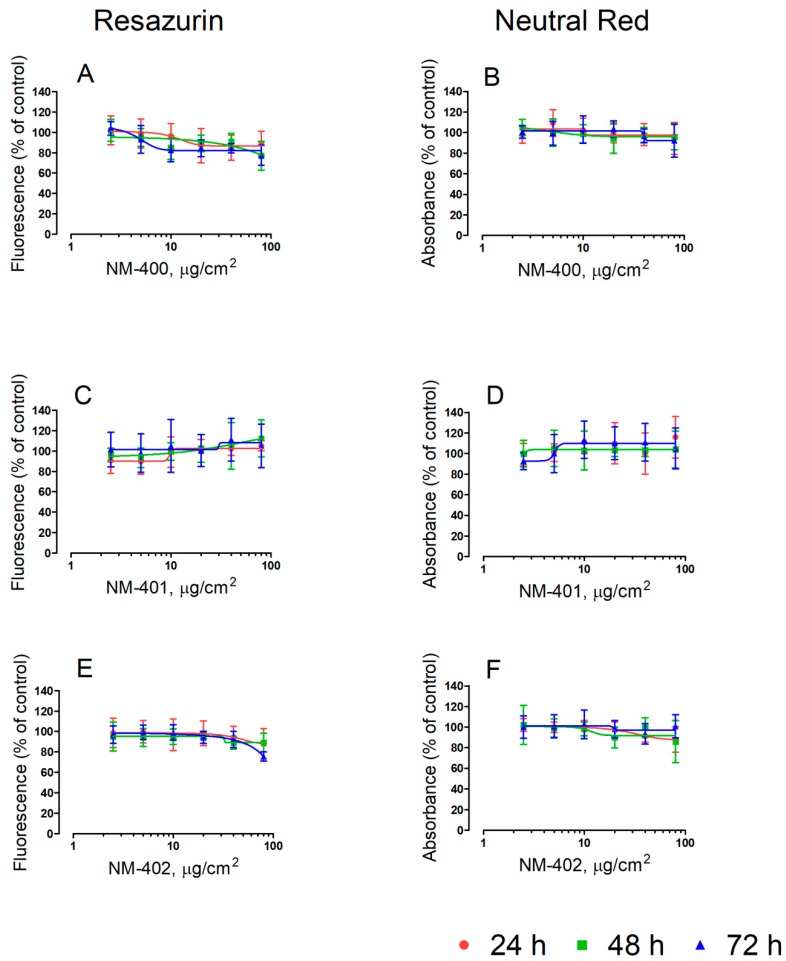
Calu-3 cells were grown for 24 h in complete growth medium and then exposed for 24 h, 48 h, and 72 h to the indicated doses of MWCNT. At the end of the incubation, cell viability was assessed with resazurin (**A**,**C**,**E**) or neutral red uptake assays (**B**,**D**,**F**). Data are the means ± SD of five independent determinations.

Several preparations of MWCNT increased the trans-epithelial permeability of human airway cell monolayers, as indicated by a significant decrease of the trans-epithelial electrical resistance [27,35,36,37]. The results shown in Figure 6 demonstrate that NM-401 caused a significant decrease in trans-epithelial resistance of Calu-3 cell monolayers grown on membrane filters. The effect was already evident at 10 μg/cm^2^ and exhibited a limited dose-dependence up to 80 μg/cm^2^ (Figure 6A). At this maximal dose, also NM-400 and NM-402 lowered trans-epithelial resistance, although the change was significantly smaller than that detected with NM-401 (Figure 6B). As expected from previously-published results [37], changes in trans-epithelial permeability were not associated with a decrease in cell viability, which was not affected by any MWCNT preparation, as assessed with the resazurin method (Figure 6C). No change in cell viability was detected in Calu-3 cell monolayers, grown on plastic dishes, after 24, 48, or 72 h of exposure to NM-400, NM-401, or NM-402 (dose range 2.5–80 μg/cm^2^; Figure 7).

## 4. Discussion

In this contribution, the effects of three preparations of MWCNT obtained from the JRC Repository were compared using macrophages and bronchial epithelial cells as cell models representative of the tissue that is expected to be firstly exposed to nanomaterials in vivo. However, since inhalation of MWCNT may lead to innate immune responses and activation of a variety of intracellular signaling cascades [38], it is important that investigations on the toxicity of inhalable nanomaterials use doses considered relevant to human occupational lifetime exposure. For this reason, we tested a broad range of occupationally-relevant doses of MWCNT. Indeed, Gangwal et al. [39] calculated the alveolar mass retained per surface area for CNT, for a working-lifetime (45 years) exposure duration. These values ranged from 12.4–46.5 µg/cm^2^, confirming that the doses implemented in our work were realistic. The three preparations differed for several physico-chemical characteristics (see Table 1) and had markedly different biological effects. NM-401 heavily affected cell viability of cultured macrophages. Viability was assessed with two independent methods, so as to minimize the risk of interference between the nanomaterial and the assay method, and in two distinct macrophage lines, so as to avoid the risk of results strictly dependent on the particular cell line used. With both methods and in both lines, NM-401 exhibited a clear-cut dose- and partially time-dependent cytotoxicity with remarkably similar IC_50_ values around 10 μg/cm^2^ (at 72 h of exposure in Raw264.7 cells). On the contrary, the other two preparations did not cause a pronounced dose-dependent acute cytotoxicity either in Raw264.7 or in MH-S macrophages. The lack of marked cytotoxicity after exposure to NM-400 and NM-402 in the latter model is remarkable, since, in a previous contribution [40], MH-S proved to be more sensitive than Raw264.7 cells to other nanomaterials. Although Calu-3 airway epithelial cells appeared much less sensitive to the toxic effects of NM-401 MWCNT than macrophages, their functional properties were profoundly affected by a prolonged (>10 d) exposure to the nanomaterials. All three MWCNT types produced a significant decrease in the trans-epithelial electrical resistance, indicative of a severe impairment of the epithelial barrier function. However, NM-401 produced a significantly larger alteration than the other two preparations even at the relatively low dose of 10 µg/cm^2^. The fact that barrier impairment occurred without evident changes in cell viability, as assessed with the resazurin method, is reminiscent of analogous findings described several years ago with other MWCNT preparations [27,35,37]. In those cases, barrier alteration was attributed to focal defects in the epithelial layer occurring next to MWCNT agglomerates. Interestingly, previously-published data [17] suggested that, once suspended in biological medium, NM-401 form agglomerates larger than NM-400 and NM-402, suggesting a relationship between barrier impairment and agglomeration tendency of the MWCNT preparation.

Evaluating the same MWCNT benchmark preparations used here in A549 and BEAS-2B human airway cells, Louro et al. [17] reported acute cytotoxic effects for NM-401, but not for the other MWCNT. However, using a clonogenic assay, the same authors described a significantly decreased proliferative capacity of A549 cells exposed to NM-400, NM-401, and NM-402 for eight days. Instead, using the resazurin assay, we did not detect any decrease in cell viability in Calu-3 cell monolayers exposed to MWCNT for 12 days. These apparently diverging effects may depend either on the greater sensitivity of the clonogenic assay compared to the resazurin method or to the fact that very dense, slowly-proliferating cultures, such as those required for TEER measurements, are usually less sensitive to toxic effects than very sparse cultures, such as those used for the clonogenic assay. Unfortunately, the TEER assay cannot be performed with A549 and BEAS-2B cells, since their monolayers do not appreciably form tight junctions [41,42]. However, it should also be stressed that TEER changes did highlight monolayer damage with all three MWCNT used, thus confirming that this assay may provide evidence for epithelial damage even if standard biochemical assays are negative [40]. Thus, viability studies would indicate substantial lack of sensitivity of airway epithelial cells to acute exposure to MWCNT, while the barrier properties of the same cell model were markedly affected upon prolonged treatment. This discrepancy indicates that in vitro testing of NM toxic effects should not simply rely on viability/cytotoxicity data.

In contrast with the enhanced biological reactivity exhibited for viability and trans-epithelial permeability, NM-401 demonstrated a lower pro-inflammatory activity on the two macrophage cell lines. Indeed, after a 48 h-exposure, both NM-400 and NM-402 induced the typical M1 marker *Nos2* and, consistently, caused a significant increase in NO output, whereas NM-401 were ineffective. Interestingly, when used to elicit acute phase response in vivo, NM-402, but not NM-400, induced higher levels of either hepatic Saa1 or pulmonary Saa3 than NM-401 [19], suggesting a higher capability to trigger an acute inflammatory response. None of the three preparations induced the M2 marker *Arg1*, indicating that, whether effective, MWCNT activated macrophages towards a M1-polarized response. However, it should be stressed that, in vivo, M2 responses require more prolonged times than M1 activation [43]. The low doses of MWCNT, used in these experiments to avoid huge, completely unrealistic, exposures, likely explain why the levels of *Nos2* expression obtained, although indicating a significant induction, were much lower than those observed with LPS, the prototypical inducer of M1 responses.

The structural determinants responsible for these biological effects may be different, and it is possible that a single physico-chemical feature responsible for the distinct behavior of NM-401 does not exist. In an ideal situation, a rigorous comparison should have been done between nanomaterial preparations differing for a single, well-characterized feature. This was not the case here, where several characteristics were different among NM-400, NM-401, and NM-402. However, some of these can be excluded on the basis of the comparable effects of preparations endowed with different properties or, conversely, on the basis of divergent effects of preparations endowed with comparable features. For example, it is unlikely that dissolution of metal contaminants justifies the different effects, since, once suspended in biological media, NM-401 did not appreciably release metals [22], and moreover, it had a Fe and Co contamination smaller than the other two preparations [22]. However, since ion release may occur in the strongly acidic endosomal environment after MWCNT internalization, a contribution of metal contaminants should not be excluded in the absence of adequate experimental evidence. The important role of redox potential is also unlikely, given that NM-402 was the only preparation to be endowed with a clear-cut and consistent ability to increase O_2_ in the suspension medium. It should also be underlined that NM-401 had the lowest specific surface area among the MWCNT tested, thus excluding that the larger viability decrease associated with the exposure to this NM may be due to the exposure to a larger dose if expressed on a surface basis. Other potential determinants of cytotoxicity have not been considered in the characterization of these materials performed by Rasmussen et al. [22]. For instance, surface chemistry has not been investigated in detail, and in particular, the possibility that subtle differences, associated with different agglomeration tendency [17,24] may change the adsorption of proteins should not be excluded. Recently, differential protein adsorption has been implied in the differential toxicological profile exhibited by pristine and functionalized MWCNT [44]. Another characteristic that may affect the effective dose per cell and the exposure kinetics is the sedimentation rate of the nanomaterials that, on the other hand, is also linked to the agglomeration tendency, which previous studies have documented to be different in the MWCNT preparations [17,24].

Among the other distinctive features of NM-400, NM-401, and NM-402, dimensional characteristics are of obvious importance. All three preparations can be defined as being high-aspect ratio nanomaterials, since the ratio between their length and diameter was well above three (79, 66, and 125 for, respectively, NM-400, NM-401, and NM-402, [22]). However, NM-401 was, on average, longer than NM-400 and NM-402. The average length of NM-401 was around 4 μm, hence slightly lower than the WHO limit for a fiber-like material (5 μm) and much lower than the MWCNT length clearly associated with frustrated phagocytosis [45,46]. However, as in most MWCNT preparations made with carbon vapor deposition, the length distribution of the NM was quite wide [22], treated cells were actually highly likely to be exposed to fiber-like (>5 μm) or even to very long MWCNT (>10 μm), which may have cause frustrated phagocytosis. Frustrated phagocytosis has been reproduced in vitro and was found associated with cell damage and cytotoxicity [47,48]. An additional, not mutually exclusive, mechanism depends on intermediate-length MWCNT, as the majority of NM-401, which may not prevent phagocytosis, but, rather, as suggested by Ji et al. for CeO nanowires [49], are phagocytosis-permissive and, once internalized and targeted to the lysosomal compartment, may produce lysosomal damage and, at later times, cytotoxicity. These combined mechanisms would be consistent with both phagocytosis impairment and loss of viability, detected in macrophages exposed to NM-401. The coexistence of the two mechanisms is favored by the presence of MWCNT of markedly different lengths in the same preparation, a characteristic that has not been often taken into consideration in toxicological studies.

Shape may also contribute to MWCNT toxic effects. NM-401 were, indeed, not only the longest MWCNT, but also the thickest among the preparations used. While the role of diameter in MWCNT cytotoxicity has been investigated in several contributions with somewhat contradictory conclusions [50,51,52], the combination of phagocytosis-permissive length and sizable thickness, causing a rigid, needle-like morphology, would favor lysosomal damage, somehow mimicking the effect of monosodium urate crystals. These mechanisms would be further enhanced by the formation of “megatubes”, due to the alignment of NM-401 in higher order structures, clearly demonstrated in the characterization study by Rasmussen et al. [22]. Conversely, NM-400 and NM-402 were thinner and formed plastic, highly-deformable agglomerates. The dimensions of these agglomerates, estimated by Louro et al. [17] under conditions comparable to those adopted in this study, were much smaller than those formed by NM-401, so that they could be internalized much more easily. It is likely that their greater efficiency in M1 macrophage activation, and hence acute inflammatory changes, depended on their better accessibility to the cell interior. However, it should be stressed that, extrapolating these results to an in vivo situation, the greater efficiency of NM-400 and NM-402 to promote acute inflammation, associated with a substantial lack of toxicity towards macrophages, would be permissive for a highly-efficient clearance of the material. On the contrary, phagocytosis impairment, epithelial barrier damage, and significant cytotoxicity, all features exhibited by NM-401-treated cells, suggest enhanced tissue bio-persistence and, possibly, the likely triggering of chronic effects. It is noteworthy that, in the in vivo study by Poulsen et al. [19], the only significant increase in the plasma levels of the inflammatory marker Saa3, documented 92 d after exposure, was found in animals exposed to NM-401 among 14 different MWCNT preparations.

## 5. Conclusions

In conclusion, using macrophage and bronchial epithelial cell lines as relevant in vitro models for inhalation exposure, this study showed that different structural features of MWCNT, and in particular length and shape, may provide a synergistic contribution to the toxicological properties of realistic doses of MWCNT. Moreover, the contribution of each structural determinant to the toxic effects is different depending on the biological system adopted and the endpoint considered. Thus, toxicology-relevant structural determinants of MWCNT should be adequately considered in the definition of human occupational exposure limits.

## Figures and Tables

**Table 1:** Main characteristics of the MWCNT preparations.

MWCNT	Length (nm) ^1^	Thickness (nm) ^1^	BET (m^2^/g) ^1^	Main Impurities ^1^ (ppm)	Redox Potential (O_2_)	Shape ^1^
NM-400	846 ± 446	11 ± 3	254.00	Na 1345 ± 151, Al 9951 ± 31, K 97 ± 3, Ca 3 ± 2, V 0, Cr 9 ± 1, Fe 1988 ± 26, Co 693 ± 26, Ni 4 ± 0, Cu 3 ± 0, Zn 2 ± 0, Ba 1 ± 0, Pb 1 ± 0 impurities wt (%) 1.41	↑/= O_2_	Highly bent (tangled)
NM-401	4048 ± 2371	67 ± 24	140.46	Na 581 ± 32, Mg 0 ± 32, Al 59 ± 4, K 57 ± 9, Ca 2 ± 1, V 1 ± 0, Cr 3 ± 1, Fe 379 ± 71, Ni 2 ± 0, Cu 3 ± 3, Zn 2 ± 1, Ba 1 ± 0 wt (%) of impurities 0.11	= O_2_	Rigid wall (needle-like)
NM-402	1372 ± 836	11 ± 3	226.4	Na 727 ± 120, Al 12955 ± 1530, K 85 ± 7, Ca 2 ± 1, V 1 ± 0, Cr 13 ± 1, Mn 9 ± 1, Fe 16321 ± 664, Co 2 ± 0, Ni 9 ± 1, Cu 4 ± 1, Zn 2 ± 0, Ba 1 ± 0 wt (%) of impurities 3.01	↑/= O_2_	Highly bent (tangled)

^1^ Data were taken from Rasmussen et al. [22].

**Table 2:** Primers used for RT-PCR studies.

Gene	Protein	Forward	Reverse	Melting Temperature (°C)
*Arg1*	Arginase I	5′-CAG AAG AAT GGA AGA GTC AG-3′	5′-GGA GTG TTG ATG TCA GTG TG-3′	49
*Nos2*	Inducible Nitric oxide synthetase (Nos2)	5′-GTT CTC AGC CCA ACA ATA CAA GA-3′	5′-GTG GAC GGG TCG ATG TCA C-3′	59
*Gapdh*	Glyceraldehyde 3-phosphate dehydrogenase	5′-TGT TCC TAC CCC CAA TGT GT-3′	5′-GGT CCT CAG TGT AGC CCA AG-3′	58
